# Supplementary material for: Uptake of cervical cancer screening and its associated factors among women of reproductive age in Kericho County
Source: PLoS One. 2026 Jan 22;21(1):e0328614. doi: 10.1371/journal.pone.0328614 (PMC12826485; doi:10.1371/journal.pone.0328614)
Supplement: Attachment — Submitted filename: Review comments.docx [file pone.0328614.s002.docx]

Summary:

This is a cross-sectional study investigating prevalence of and factors associated with cervical cancer among women of reproductive age (N=328) in Kericho County, Kenya. Authors used a systematic random sampling approach to select participants from one hospital. Data was collected using questionnaires and regression performed to assess the hypothesized associations.

Abstract:

1. Consider rephrasing <of which 85%> to 85% of which ….

Background

1. It is the leading cause of all cancer deaths in Kenya, with over 3,200 deaths in 2020 (2) -> this data is outdated. Consider more recent statistics and cite here
2. Most scholars -> consider changing to “experts”
3. This is alarming given that Kenya has been implementing a national screening program in the past 10 years. -> citation missing
4. Additionally, with the high level of awareness among Kenyan women reported by it is expected that uptake of cancer screening should be high -> grammar, check for additional /missing words here.
5. Thus, there could be other factors contributing to the low screening uptake. Therefore, this study aimed to assess the prevalence and factors associated with cervical cancer screening among women aged 18-49 years in Bureti Constituency, Kericho County. -> this sentence could be further strengthened to illustrate the gap in research

Study design

1. located in the rural setting. -> consider changing to : ….a rural setting
2. Sociodemographic section entailed 11 items asked questions -> (consider editing to): The sociodemographic section entailed 11 items (and) asked questions …
3. Consider editing “Those who scored a cut off of 50% and above were regarded to have good knowledge while those who failed were regarded poor.” -> Here’s a cleaner revision of your sentence: Those who scored **50% or above** were considered to have good knowledge, while those who scored below 50% were **considered** to have poor knowledge.
4. Consider rephrasing: …to -> out of 328 respondents, 48 (33.1%) were aged 18–24 years; the majority were Christians (97.2%), married (50.3%), had attained secondary education (34.5%), reported business as their main source of income (39.3%), and earned less than Ksh. 2,000 (46.9%).

**Association between sociodemographic factors and cervical cancer screening**

1. **“…..**compared to those who aged 18–24 years.” -> consider rephrasing to compared to those aged 18–24 years

**Discussion**

1. Individuals who earned a total monthly household income Ksh. 2, 000 – 5, 000 (aOR: 0.36, 95% CI: 0.063 – 0.79, p = 0.011) and more than Ksh. 10, 000 (aOR: 0.13, 95% CI: 0.059 – 0.307, p < 0.001) were less likely to be screened compared to counterparts earning less than Ksh. 2, 000.
2. Family history of cervical cancer is significantly -> .. “was significantly..”
3. According to Kenyan Demographic and Health Survey 2022 report reported that a national prevalence of cervical cancer screening of 16.7%, -> The Kenyan Demographic and Health Survey 2022 report showed a national…..
4. “symptoms of cervical cancer cervical cancer, and knowledge on groups at risk were less likely to screen” -> grammar error
5. Studies have reported that good knowledge of cervical cancer risk factors and signs and symptoms increase likelihood of screen uptak -> grammar
6. and groups at risk of cervical cancer significantly impact utilization its screening. T-> grammar
