# Supplementary material for: Uptake of cervical cancer screening and its associated factors among women of reproductive age in Kericho County
Source: PLoS One. 2026 Jan 22;21(1):e0328614. doi: 10.1371/journal.pone.0328614 (PMC12826485; doi:10.1371/journal.pone.0328614)
Supplement: Attachment — Submitted filename: Rebattal letter.docx [file pone.0328614.s004.docx]

**REBATTAL LETTER**

**REVIEWER 1**

| Abstract |  |
| --- | --- |
| 1. Consider rephrasing <of which 85%> to 85% of which | Revised as advised |
| Background |  |
| 1. It is the leading cause of all cancer deaths in Kenya, with over 3,200 deaths in 2020 (2) -> this data is outdated. Consider more recent statistics and cite here | There was none, so the sentence was deleted |
| 2. Most scholars -> consider changing to “experts”…………… | Changed |
| 3. This is alarming given that Kenya has been implementing a national screening program in the past 10 years. -> citation missing | Cited |
| 4. Additionally, with the high level of awareness among Kenyan women reported by it is expected that uptake of cancer screening should be high -> grammar, check for additional /missing words here. | rewritten as “Based on Gebreegziabher's report, the high level of awareness among Kenyan women suggests that cancer screening uptake should be high” |
| 5. Thus, there could be other factors contributing to the low screening uptake. Therefore, this study aimed to assess the prevalence and factors associated with cervical cancer screening among women aged 18-49 years in Bureti Constituency, Kericho County. -> this sentence could be further strengthened to illustrate the gap in research …….. | rewritten as “While awareness of cervical cancer is reportedly high among Kenyan women, the reasons for the consistently low screening uptake remain largely” |
| Study design |  |
| 1. located in the rural setting. -> consider changing to : ….a rural setting…… | Revised as advised |
| 2. Sociodemographic section entailed 11 items asked questions -> (consider editing to): The sociodemographic section entailed 11 items (and) asked questions | Revised as advised |
| 3. Consider editing “Those who scored a cut off of 50% and above were regarded to have good knowledge while those who failed were regarded poor.” -> Here’s a cleaner revision of your sentence: Those who scored **50% or above** were considered to have good knowledge, while those who scored below 50% were **considered** to have poor knowledge………. | Revised as advised |
| **Results** |  |
| 1. Consider rephrasing: …to -> out of 328 respondents, 48 (33.1%) were aged 18–24 years; the majority were Christians (97.2%), married (50.3%), had attained secondary education (34.5%), reported business as their main source of income (39.3%), and earned less than Ksh. 2,000 (46.9%) | Revised as advised |
| **Association between sociodemographic factors and cervical cancer screening**   2. “…..compared to those who aged 18–24 years.” -> consider rephrasing to compared to those aged 18–24 years | Revised as advised |
| **Discussion** |  |
| 1. Individuals who earned a total monthly household income Ksh. 2, 000 – 5, 000 (aOR: 0.36, 95% CI: 0.063 – 0.79, p = 0.011) and more than Ksh. 10, 000 (aOR: 0.13, 95% CI: 0.059 – 0.307, p < 0.001) were less likely to be screened compared to counterparts earning less than Ksh. 2, 000 | Revised as advised |
| 2. Family history of cervical cancer is significantly -> .. “was significantly..” | Revised as advised |
| 3. According to Kenyan Demographic and Health Survey 2022 report reported that a national prevalence of cervical cancer screening of 16.7%, -> The Kenyan Demographic and Health Survey 2022 report showed a national | Revised as advised |
| 4. “symptoms of cervical cancer cervical cancer, and knowledge on groups at risk were less likely to screen” -> grammar error | Revised |
| 5. Studies have reported that good knowledge of cervical cancer risk factors and signs and symptoms increase likelihood of screen uptak -> grammar | Revised |
| 6. and groups at risk of cervical cancer significantly impact utilization its screening. T-> gramma | Revised |

**REVIEWER 2**

| The manuscript is presented clearly and concisely. The following are specific review comments, with the first two being minor and the last one being major. |  |
| --- | --- |
| **Specific comments** |  |
| 1. The abstract results have to be presented uniformly. Some of the factors indicated toward the end do not have the respective OR and p-values like the prior variables. | Revised as advised |
| 2. The way the cross-tabulation tables were presented was misleading, and I would suggest that the authors do row percentages to identify the association of factors on the magnitude of screening. | Revised as advised |
| 3. Why are two models needed for the particular outcome variable? There should be a final model that takes into consideration variables significant in both models. The authors can organize them as models I, II, and III. | Revised as advised |
